# The pattern of gene copy number alteration (CNAs) in hepatocellular carcinoma: an in silico analysis

**DOI:** 10.1186/s13039-021-00553-2

**Published:** 2021-07-02

**Authors:** Arman Shahrisa, Maryam Tahmasebi-Birgani, Hossein Ansari, Zahra Mohammadi, Vinicio Carloni, Javad Mohammadi Asl

**Affiliations:** 1 Department of Genetics, Faculty of Biological Sciences, Tarbiat Modares University, Tehran, Iran; 2 Department of Medical Genetics, School of Medicine, Ahvaz Jundishapur University of Medical Sciences, Ahvaz, Iran; 3 Cellular and Molecular Research Center, Medical Basic Sciences Research Institute, Ahvaz Jundishapur University of Medical Sciences, Ahvaz, Iran; 4 Department of Biotechnology, Islamic Azad University, Ahvaz Branch, Ahvaz, Iran; 5 School of Medicine, Shiraz University of Medical Sciences, Shiraz, Iran; 6 Department of Experimental and Clinical Medicine, University of Florence, Florence, Italy

**Keywords:** Hepatocellular carcinoma, Copy number, RNA dysregulation, chr8p, chr1q

## Abstract

**Background:**

Hepatocellular carcinoma (HCC) is the most common type of liver cancer that occurs predominantly in patients with previous liver conditions. In the absence of an ideal screening modality, HCC is usually diagnosed at an advanced stage. Recent studies show that loss or gain of genomic materials can activate the oncogenes or inactivate the tumor suppressor genes to predispose cells toward carcinogenesis. Here, we evaluated both the copy number alteration (CNA) and RNA sequencing data of 361 HCC samples in order to locate the frequently altered chromosomal regions and identify the affected genes.

**Results:**

Our data show that the chr1q and chr8p are two hotspot regions for genomic amplifications and deletions respectively. Among the amplified genes, *YY1AP1* (chr1q22) possessed the largest correlation between CNA and gene expression. Moreover, it showed a positive correlation between CNA and tumor grade. Regarding deleted genes, *CHMP7* (chr8p21.3) possessed the largest correlation between CNA and gene expression. Protein products of both genes interact with other cellular proteins to carry out various functional roles. These include ASH1L, ZNF496, YY1, ZMYM4, CHMP4A, CHMP5, CHMP2A and CHMP3, some of which are well-known cancer-related genes.

**Conclusions:**

Our in-silico analysis demonstrates the importance of copy number alterations in the pathology of HCC. These findings open a door for future studies that evaluate our results by performing additional experiments.

## Background

Hepatocellular carcinoma (HCC) is the most common type of liver cancer that occurs predominantly in patients with chronic liver disease and cirrhosis. The well-established risk factors of HCC are hepatitis B virus infection, alcoholism and metabolic disorders [1, 2]. Although liver lesions are usually detectable on computed tomography (CT), many HCC tumors are asymptomatic. As a result, HCC is usually diagnosed after development of end-stage clinical symptoms [3]. Despite the numerous researches, no effective screening modality exists for HCC. This necessitates identifying the key genetic factors and new potential therapeutic targets. However, this is a complicated process because of the heterogeneous nature of HCC [4].

Recent findings demonstrate that Copy number alteration (CNA) can lead to activation of oncogenes and inactivation of tumor suppressor genes in various cancers, which in turn predispose the cells toward malignancy. These genetic alterations include various chromosomal losses or gains that can cause the genome copy number of the affected cells to deviate from normal diploid state [5, 6]. Subsequently, this affects the genome stability and drives the cells toward tumorigenesis [7]. Chromosomal aberrations are associated with poor prognosis and function (age of onset, metastatic state, drug resistant and liver failure phenotypes) [8]. Therefore, it is helpful to identify the hotspot chromosomal region at which these genetic alterations are concentrated as well as the list of the genes that are affected by these alterations [9].

Recent advances in functional genomics along with the invention of comparative genomic hybridization (CGH) and microarray-based techniques permit the scientists to characterize the cytogenetic signatures of the malignant cells. The emergence of bioinformatic tools adds another level of proficiency that is needed for translating high-throughput data to improve cancer research, speeding up the discovery of hundreds of new potential targets, and finally, facilitating the developments of new prognostic, diagnostic and therapies approaches [10, 11]. Here, by means of an in silico analysis, we have evaluated the patterns of CNA in liver HCC samples to identify hotspot chromosomal regions that are altered during the course of disease. Our research provides the preliminary support for further laboratory experiments.

## Results

### Chromosomal cytobands chr1q and chr8q were strongly associated with gene amplification in HCC samples

**Fig. 1:**
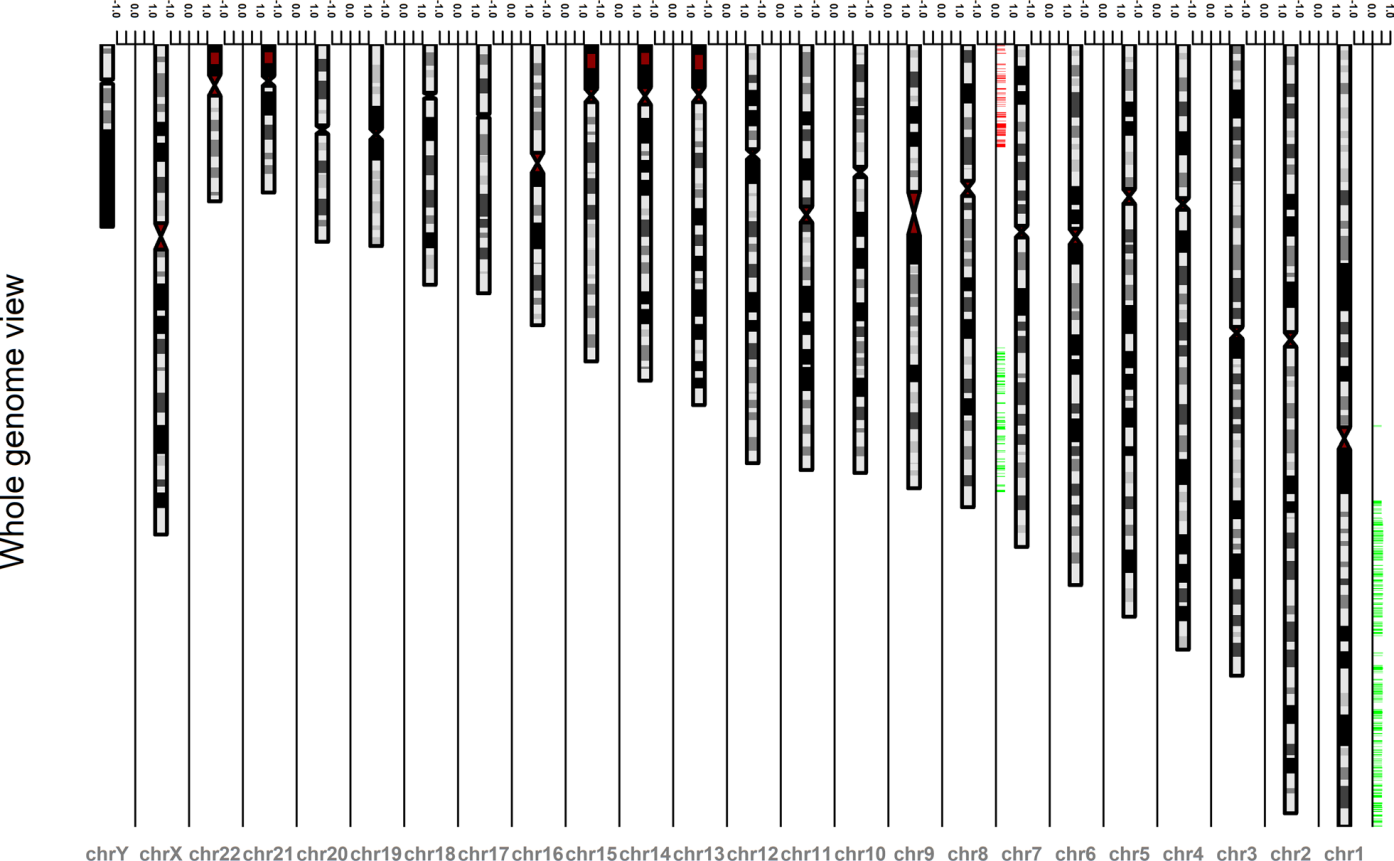
Human chromosome ideograms showing regions of copy number variation (deletion and/or amplification) in a high resolution (244 K) whole genome hybridization (aCGH) analysis of 361 subjects with HCC. Deletions are shown in red and amplification in green. Data was extracted from cBioPortal dataset (https://www.cbioportal.org) through Bioconductor package cgdsr and the ideogram was generated by Bioconductor package IdeoViz

**Fig. 2:**
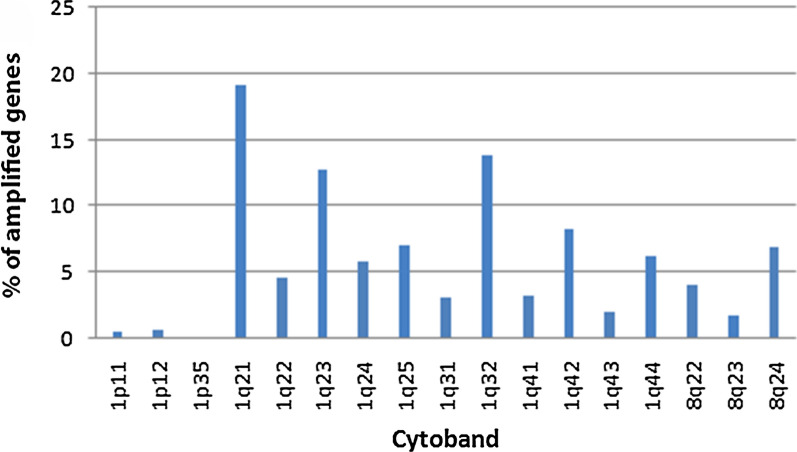
Presentation of hotspot regions for all amplified genes on chr 1 and chr 8. 361 HCC samples posessing both expression data as well as CNV data were used to draw this plot. Data was extracted from cBioPortal dataset (https://www.cbioportal.org)

Of 24,776 examined genes, 1024 (4.1%) were amplified in HCC samples (linear copy number values cut-off ≥ 0.5). These genes were mapped on chr1q (86%; n = 880), chr8q (13%; n = 131) and chr1p (1.3%; n = 13) (Fig. 1). Figure 2 shows the frequency with which different cytobands are observed for all the 1024 amplified genes. The top 34 genes with the high amplification scores were *ADAM15, FLAD1, ZBTB7B, PYGO2, DCST2, LENEP, SHC1, PBXIP1, PMVK, DCST1, KCNN3, SLC50A1, DPM3, SCAMP3, FAM189B, MTX1, KRTCAP2, GBA, GBAP1, MUC1, THBS3, TRIM46, ASH1L, RUSC1, CLK2, HCN3, FDPS, PKLR, YY1AP1, MSTO1, GON4L, RIT1, SYT11* and *KIAA0907* (Table 1)*.* Majority of these 34 amplified genes are located on chr1q22 (67.6%; n = 23) while the remaining are on chr1q21.3 (32.4%; n = 11). Amplification of 13 genes were associated with tumor grade (Table 2). However, we observed no association between gene amplification and other clinicopathologic parameters, such as tumor size, stage and metastasis. Although all the 34 selected genes were amplified in HCC samples, we only observed a strong correlation between the CNA of oncogene *YY1AP1* (Yin Yang-1 Associated Protein 1) and its expression (72% of studied samples; r > 0.6). PANTHER classified the protein products of these 34 genes in 15 cellular processes (Table 1), with many of them being well-recognized signaling pathways. These pathways include Wnt signaling pathway, angiogenesis signaling pathway, Ras signaling pathways, EGF signaling pathway, FGF signaling pathway, interleukin signaling pathway, and PDGF signaling pathway. We also noticed the involvement of some other pathways in HCC that are less emphasized in literature, if not completely ignored. Around 12.5% of these proteins are associated with cholesterol biosynthesis. Other pathways include flavin signaling pathway, glycolysis pathway, inflammation mediated by chemokine and cytokine signaling pathway, integrin signaling pathway, pyruvate metabolism pathway, and synaptic vesicle trafficking.

**Table 1:** The role of 34 amplified genes and 17 deleted genes with the highest scores according to PANTHER (http://pantherdb.org/)

Gene name	Protein class	Pathway(s) involved
Amplified genes		
*ADAM15*	Metalloprotease	–
*FLAD1*	Transferase	Flavin biosynthesis
*ZBTB7B*	–	–
*PYGO2*	–	Wnt signaling pathway
*DCST2*	–	–
*LENEP*	–	–
*SHC1*	Scaffold/adaptor protein	Inflammation mediated by chemokine and cytokine signaling pathway PDGF signaling pathway FGF signaling pathway CCKR signaling map Angiogenesis EGF receptor signaling pathway Integrin signaling pathway Ras Pathway Interleukin signaling pathway
*PBXIP1*	–	–
*PMVK*	Kinase	Cholesterol biosynthesis
*DCST1*	–	–
*KCNN3*	Voltage-gated ion channel	–
*SLC50A1*	–	–
*DPM3*	Transferase	–
*SCAMP3*	Transfer/carrier protein	–
*FAM189B*	–	–
*MTX1*	–	–
*KRTCAP2*	–	–
*GBA*	–	–
*GBAP1*	–	–
*MUC1*	Cell adhesion molecule	–
*THBS3*	–	–
*TRIM46*	Ubiquitin–protein ligase	–
*ASH1L*	Histone modifying enzyme	–
*RUSC1*	–	–
*CLK2*	Non-receptor serine/threonine protein kinase	–
*HCN3*	Ion channel	–
*FDPS*	Acyltransferase	Cholesterol biosynthesis
*PKLR*	Kinase	Glycolysis Pyruvate metabolism
*YY1AP1*	Transcription cofactor	–
*MSTO1*	–	–
*GON4L*	Transcription cofactor	–
*RIT1*	Small GTPase C2H2 zinc finger transcription factor	–
*SYT11*	Membrane trafficking regulatory protein	Synaptic vesicle trafficking
*KIAA0907*	–	–
Deleted genes		
*TNFRSF10B*	Transmembrane signal receptor	Apoptosis signaling pathway
		p53 pathway
*RHOBTB2*	Small GTPase	–
*PEBP4*	Protease inhibitor	FGF signaling pathway
		EGF receptore signaling pathway
*TNFRSF10C*	Transmembrane signal receptor	Apoptosis signaling pathway
*CHMP7*	Membrane traffic protein	–
*TNFRSF10A*	Transmembrane signal receptor	Apoptosis signaling pathway p53 pathway
*ENTPD4*	Nucleotide phosphatase	–
*EGR3*	C2H2 zinc finger transcription factor	–
*BIN3*	–	–
*PDLIM2*	Actin or actin-binding cytoskeletal protein	–
*R3HCC1*	Unknown	–
*LOXL2*	Oxidase	–
*STC1*	Peptide hormone	–
*PIWIL2*	Translation initiation factor	
*SLC25A37*	Unknown	–
*TNFRSF10D*	Transmembrane signal receptor	Apoptosis signaling pathway p53 pathway
*CSMD1*	–	–

**Table 2:** The association of copy number variations with clinicopathologic parameters of 361 HCC samples. Only candidate genes with significant association have been presented in the table

Gene symbols (n = 13)	CNA type	Clinical parameters
*YY1AP1, FLAD1, FAM189B, DCST2, ZBTB7B, TRIM46, SYT11, SHC1, SCAMP3, RIT1, LENEP, MSTO1, KIAA0907*	AMP	Tumor grade

**Fig. 3:**
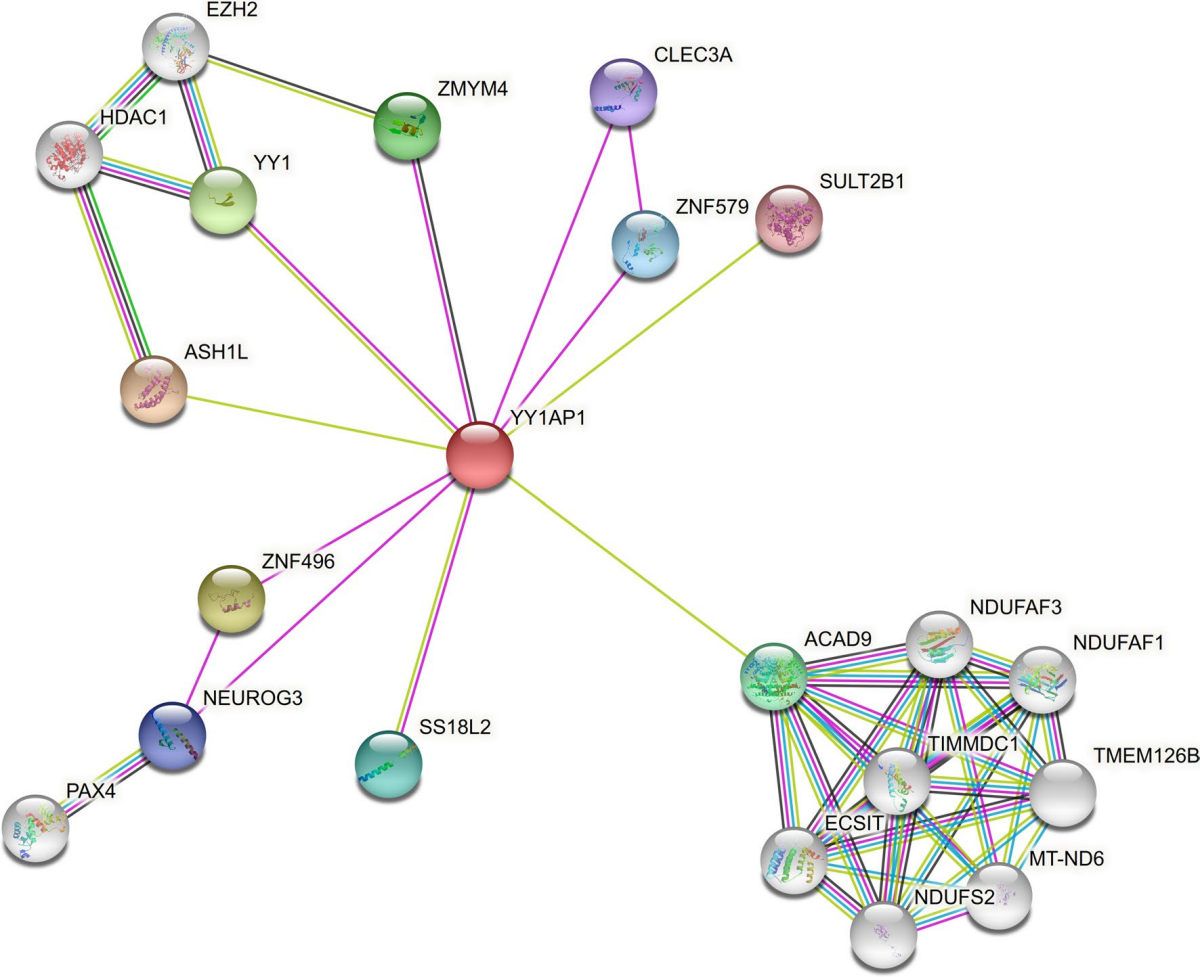
The top protein intractions with YY1AP1 protein, obtained from STRING version 11 (r > 0.5; cyan = from curated databases; pink = experimentally determined; olive = textmining; dark azure = gene co-occurrence; black = co-expression)

Analysis of protein interaction networks predicts 21 protein–protein interactions involving YY1AP1 and other proteins either directly or through intermediary. The top five interacting partners with scores greater than 0.6 are ASH1L, ZNF496 (Zinc Finger Protein 496), YY1 (Yin Yang 1), ZMYM4 (Zinc Finger MYM-Type Containing 4) and ACAD9 (Acyl-CoA dehydrogenase family member 9) (Fig. 3). Functional analysis shows that some of these genes belong to critical cellular components such as Nucleolar Remodeling Complexes (NoRC) that negatively regulates rRNA expression, and transcriptional machinery (Table 3).

**Table 3:** The functional role of proteins interacting with YY1AP1 and CHMP7 according to g:profiler

Term ID	Term name	p_adj_
*YY1AP1*-interacted proteins		
CORUM:3046	hs4 enhancer complex (slow migrating complex)	4.995 × 10^–2^
GO:0033202	DNA helicase complex	3.190 × 10^–3^
GO:0031011	Ino80 complex	3.190 × 10–3
GO:0070603	SWI/SNF superfamily-type complex	8.722 × 10^–4^
GO:0097346	INO80-type complex	9.303 × 10^–3^
GO:1904949	ATPase complex	2.234 × 10^–3^
*CHMP7-*interacted proteins		
REAC:R-HSA-917729	Endosomal Sorting Complex Required For Transport (ESCRT)	1.091 × 10^–21^
REAC:R-HSA-162588	Budding and maturation of HIV virion	1.769 × 10^–18^
GO:1904896	ESCRT complex disassembly	1.492 × 10^–19^
GO:1902410	Mitotic cytokinetic process	1.009 × 10^–19^
GO:0071985	Multivesicular body sorting pathway	6.212 × 10^–21^
GO:0061952	Midbody abscission	5.004 × 10^–21^
GO:0046755	Viral budding	7.103 × 10^–23^
GO:0045324	late endosome to vacuole transport	1.513 × 10^–19^
GO:0036257	Multivesicular body organization	5.573 × 10^–26^
GO:0032509	Endosome transport via multivesicular body sorting pathway	1.192 × 10^–21^
GO:0019068	Virion assembly	6.212 × 10^–21^
GO:0007034	Vacuolar transport	1.928 × 10^–18^

MicroRNA target prediction also identified two miRNAs—*hsa-miR-375* and *hsa-miR-222-3p*—that target YY1AP1, thereby they are able to regulate the concentration of YY1AP1 protein in the cell (Table 4).

**Table 4:** Human miRNAs target human YY1AP1 and CHMP7. Data were extracted from mirWalk dataset (http://mirtarbase.cuhk.edu.cn/php/search.php)

ID	miRNA	Target
MIRT004478	hsa-miR-375	YY1AP1
MIRT046762	hsa-miR-222-3p	YY1AP1
MIRT030249	hsa-miR-26b-5p	CHMP7
MIRT037936	hsa-miR-505-5p	CHMP7
MIRT042391	hsa-miR-484	CHMP7
MIRT046411	hsa-miR-15b-5p	CHMP7

### Cytoband chr8p was strongly associated with gene deletion in HCC samples

**Fig. 4:**
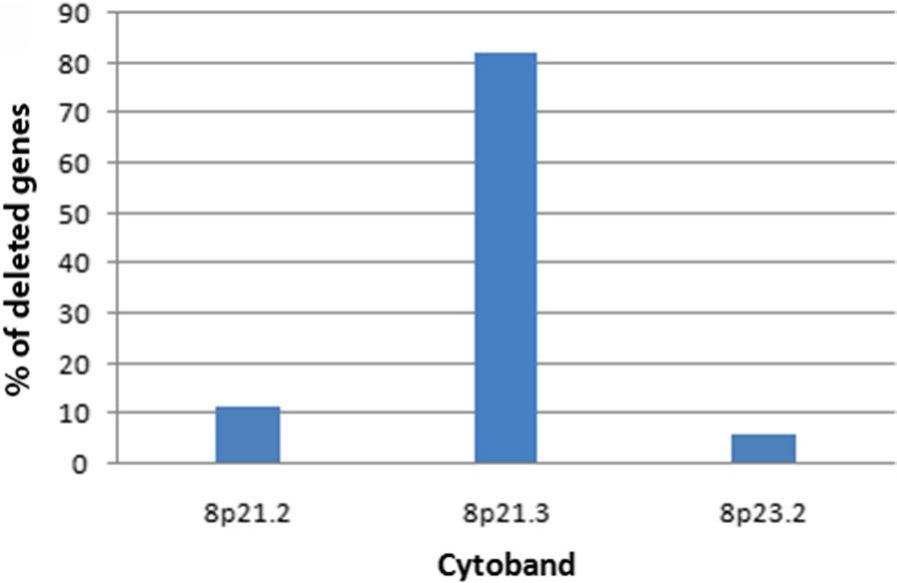
The frequency of cytogenetic bands involved in all deleted genes on chromosme 8. 361 HCC samples posessing both expression data as well as CNV data were used to draw this plot. Data were extracted from cBioPortal dataset (https://www.cbioportal.org)

Of 24,776 examined genes, 172 (0.69%) were lost in samples (n = 361) (linear copy number values cut-off ≥ 0.5). Majority of these genes are located on chr8p21-23 (Fig. 1). We selected the top 17 genes with the high deletion scores (Table 1)—*TNFRSF10B, RHOBTB2, PEBP4, TNFRSF10C, CHMP7, TNFRSF10A, ENTPD4, EGR3, BIN3, PDLIM2, R3HCC1, LOXL2, STC1, PIWIL2, SLC25A37, TNFRSF10D* and *CSMD1—*for further analysis*.* Among these 17 genes, 16 are on chr8p21.3 (94%) and one on chr8p23.2 (6%) (Fig. 4). These genes encode proteins that function as either transcription factors or signaling/cytoskeletal proteins and take part in several processes including EGF, FGF, apoptosis, and P53 signaling pathways (Table 1). We didn’t observe any strong association between deletions of these 17 genes with tumor metastasis or grade. Although all the mentioned genes were downregulated in studied cancerous samples, only *CHMP7* (Charged Multivesicular Body Protein 7) gene showed a moderate correlation between CNA and expression values (r = 0.5) in 70% of studied samples.

**Fig. 5:**
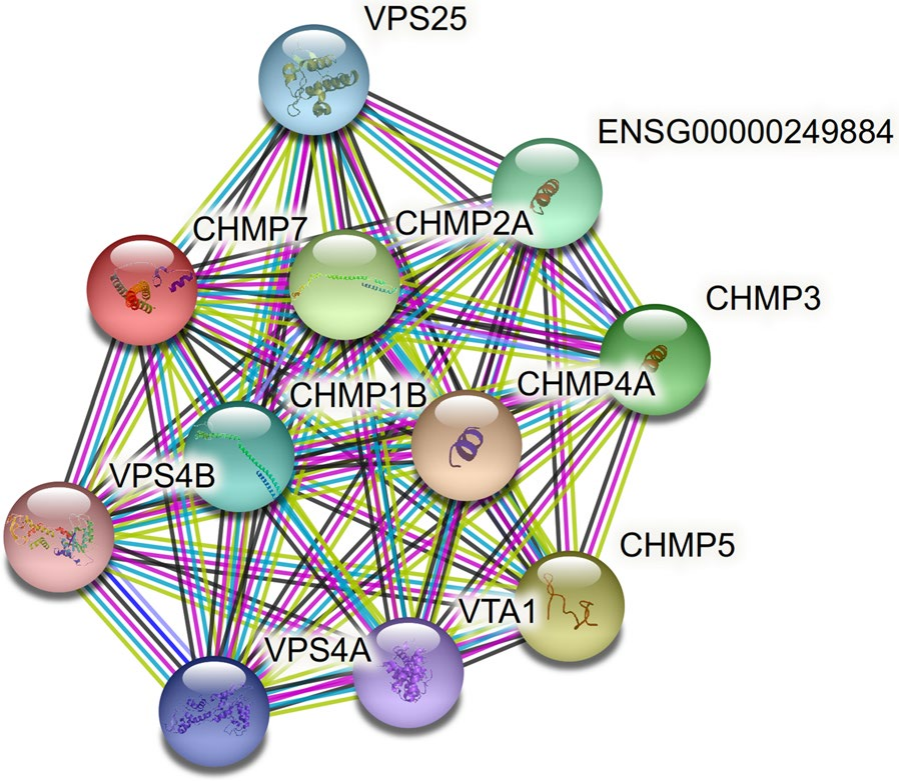
The top protein interactions with CHMP7, obtained from STRING version 11 (r > 0.9; cyan = from curated databases; pink = experimentally determined; olive = textmining; dark azure = gene co-occurrence; black = co-expression)

STRING database predicts direct protein–protein interactions between the CHMP7 protein and ten other proteins, among which the top five interactions with scores greater than 0.9 are with CHMP4A, CHMP5, CHMP2A, CHMP3 and ENSG00000249884 (*RNF103-CHMP3* gene) (Fig. 5). According to functional analysis, some of these genes belong to vital cellular pathways including endosome transport, cytokinesis, vacuolar transport and viral assembly. (Table 3).

MicroRNA target prediction also recognized four miRNAs *hsa-miR-26b-5p*, *hsa-miR-505-5p*, *hsa-miR-484* and *hsa-miR-15b-5p* that target CHMP7 to influence its concentration in the cell (Table 4).

## Discussion

We identified two hotspot regions chr1q and chr8p as locations that carry most of the chromosomal gains and losses respectively. This finding is in agreement with results from earlier studies. Comparative genomic hybridization on hepatocellular carcinoma samples shows involvement of both of these chromosomal regions, among several other regions [12]. Takafumi Nishimura et al*.* also noticed that one of the most common features of HCC is the recurrent chromosomal gain at chr1q [13]. Besides, Chen and coworkers discovered that chr1q and chr8p are among the frequently occurring gains and losses in HCC respectively [14]. Other independent studies have also highlighted the involvement of chr8p cytoband in HCC [15, 16]. Intriguingly, Yosuke Kishimoto et al. claim that HCC may develop from cirrhotic cells carrying chr8p loss [17].

We were also able to identify list of the genes that are influenced by these copy number alterations. Majority of these amplified and deleted genes are involved in various pathways, several of which are repeatedly reported as critical routs in HCC [18, 19]. Whereas amplified genes were located on either chr1q, chr1p or chr8q, deleted genes were located on chr8p. We noticed that 13 out of top 34 amplified genes are associated with tumor grade. Tae-Min Kim et al. have also found that gain in 8q11.21-q24.3 is associated with the high tumor grade [5]. On the other hand, Xin‐Yuan Guan et al. have reported an association between gain of 1q and 8q and stage of hepatocellular carcinoma [20] and Shirley M-H Sy and colleagues suggested a higher incidence of regional 1q21–q22 in association with the advanced stage tumors [21]. Despite the reported association of these chromosomal regions with tumor stage in general, we didn’t find any significant association between amplification of selected 34 genes and tumor stage.

Combing the RNA-seq and CNA data allowed us to identify subset of these genes that are simultaneously affected by both copy number alterations and gene expression pattern. Around 70% of the samples with gain of *YY1AP1* or loss of the *CHMP7* were also upregulated or downregulated respectively. YY1AP1 protein is a component of the INO80 chromatin remodeling complex, which is responsible for transcriptional regulation, DNA repair, and replication [22]. YY1AP1 acts as a critical oncoprotein in HCC by promoting the cell proliferation; maintaining stem cell features and epigenetically regulating of transcriptional networks. Moreover, silencing YY1AP1 eliminates the oncogenic properties of HCC cells by altering the chromatin structure and triggers apoptosis in vitro [4]. Sela et al. have also noticed that *YY1AP1* possess exons that are intronic within normal tissues and are recognized as exons only within cancerous tissues. These exons are known as *Alu* exons [23] which are derived from *Alu* elements: the most common transposable element in the primate genomes [24]. This would explain the significance of *YY1AP1* as a gene with both altered copy number and expression values.

Interestingly of five top proteins that interact with YY1AP1, four—ASH1L, ZNF496, YY1 and ZMYM4—are either transcription factors or transcriptional activators, while ACAD9 is involved in the beta-oxidation of fatty acyl-CoA in mitochondria. ASH1L is a histone methyltransferase that catalyzes the dimethylation and trimethylation of H3K36 and therefore is a transcriptional activator [25]. Silencing *ASH1L* through RNAi increases the sensitivity of HepG2 cells to sorafenib, suggesting that ASH1L may play a role in drug resistance in HCC cells [26]. In lung cancer, ASH1L also stimulates migration of cancer cells through Cdk5/p35 pathway [27]. YY1 is a famous oncogene widely involved in the development of many cancers including hepatocellular carcinogenesis. YY1AP1 acts as a coactivator of YY1 transcription factor [4]. YY1 is a critical component of epigenetic regulatory networks that dictates progression of hepatocellular carcinoma and suppresses the differentiation of hepatocytes [28, 29]. Both ZMYM4 (Zinc Finger MYM-Type Containing 4) and ZNF496 (Zinc finger protein 496) are identifiable from serum samples of patients with HCC [30]. Unfortunately, we don’t know anything about their function apart from the fact that they are zinc finger proteins overexpressed in multiple cancers [31, 32].

Adding to the complexity of interactions, two microRNAs can target *YY1AP1* with opposite consequences. While hsa-miR-375 generally has a tumor suppressor role [33], hsa-miR-222-3p shows oncogenic properties [34].

Considering all the aspects, YY1AP1 is a key player in HCC with complicated transcriptional and epigenetic roles, exerting its effect either directly or through binding partners. This scheme is consistent with our finding that YY1AP1 shows significant changes in both copy number and gene expression data.

CHMP7 is a key component of ESCRT-III machinery that is involved in membrane abscission during cytokinesis, endosomal sorting, plasma membrane repair and nuclear envelope reforming [35]. Although, there are no publication regarding the importance of *CHMP7* gene in HCC, activation of ESCRT-III machinery leads to membrane repair by shedding damaged parts of cell membranes. ESCRT-III is associated with resistance to cell death, especially when treated with anticancer agents [36].

In the same way as CHMP7, interacting proteins CHMP4A, CHMP5, CHMP2A, CHMP3 are components of mammalian ESCRT-III endosomal sorting complex while ENSG00000249884 is the result of readthrough transcription of CHMP3 and the adjacent gene RNF103. Apart from CHMP4A, the rest of these proteins show changes in their expression pattern in HCC [37, 38]. Our functional analysis suggests that CHMP7 and its interacting proteins promotes mitotic cytokinetic, midbody abscission and multivesicular body organization. Interestingly, some of these functional roles involve viral infection, which might explain the significance of ESCRT-III complex in progression of HCC. Infection by hepatitis B and C viruses is a major cause of hepatocellular carcinoma [39, 40]. Both of these viruses are dependent to ESCRT-III complex for their production, maturation and releases [41–43]. In the TCGA HCC dataset that we studied, 46% of total patients were in stage I alone, with less than 0.1% of them in late stage IV, suggesting that down-regulation of ESCRT-III is happening mostly at early stages of HCC but its significance is unclear.

We also found that four microRNAs target CHMP7: hsa-miR-26b-5p, hsa-miR-505-5p, hsa-miR-484 and hsa-miR-15b-5p. hsa-miR-26b-5p is involved in hepatitis B virus mediated HCC [44]; hsa-miR-484 shows both oncogenic and tumor-suppressor properties depending on the interacting partners [45, 46] and miR-15b-5p is a potential tumor suppressor [47, 48]. Altogether, the complex network of interactions suggests that CHMP7 could play important role(s) in mediating the HCC.

## Conclusions

We combined RNA-seq and copy number data for hepatocellular carcinoma. Copy number alteration mainly affected the chromosomal regions chr1q and chr8p, mostly influencing 34 genes with gains and 17 genes with losses. These genes are linked to various pathways that are famous for their role in HCC. Among these, *YY1AP1* and *CHMP7* genes demonstrated significant changes in both copy number and gene expression data. Considering these findings, we suggest that candidate regions chr1q and chr8p in HCC could be subjects of further researches, with a major emphasis on the role of two genes *YY1AP1* and *CHMP7*.

## Methods

As chromosomal CNAs may affect the level of gene expression, the CNA and RNA-seq data were analyzed in parallel. The data for Liver Hepatocellular Carcinoma (TCGA, Provisional) possessing 24,777 genes and 361 samples were downloaded from cBioPortal [49, 50] through the “cgdsr” package [51] and analyzed in R v3.5. The CNA data were filtered by considering the linear copy number values cut-off ≥ 0.5 and cut-off ≤ -0.5 as thresholds for gene amplification and deletion respectively. The filtered results were further examined to identify the genes with significant correlation between their CNA and expression values. As in strong positive correlation, the linear correlation coefficient (r) is close to + 1, the results were filtered based on the r > 0.7.

The association of CNV variants with pathological tumor grade and stage was examined in R. The class type of selected proteins and their involvement in various pathways were estimated using the PANTHER classification system [52]. STRINGDB [53] was employed to identify the proteins that have functional and physical interactions with genes that possess both significant CNA and expression changes. Involvement of these proteins as groups in various biological processes were assessed by g:Profiler [54]. MiRWalk [55] was used to predict the presence of seed sequence in the selected genes. Two databases, one for oncogenes (http://ongene.bioinfo-minzhao.org/) and one for tumor suppressor genes (https://bioinfo.uth.edu/TSGene/), helped to identify if any of the selected genes are listed among oncogenes/tumor suppressor genes.

## Data Availability

The datasets used and/or analyzed during the current study are available on cbioportal.org and are also accessible through cgdsr R package.
